# Design and experiment of a soybean shaftless spiral seed discharge and seed delivery device

**DOI:** 10.1038/s41598-023-48117-1

**Published:** 2023-11-25

**Authors:** Huibin Zhu, Xian Wu, Lizhen Bai, Rongdong Li, Guanyu Guo, Jin Qin, YuanYuan Zhang, Hui Li

**Affiliations:** 1 Faculty of Modern Agricultural Engineering, Kunming University of Science and Technology, Kunming, 650500 China; 2 Shandong Academy of Agricultural Machinery Science, Ji’nan, 250100 China

**Keywords:** Engineering, Mechanical engineering

## Abstract

When the driven stubble-breaking and anti-blocking no-till planter operates in the Southwest China, the stubble-breaking blades will impact with the ground as they cut through the soil and straw stubble, causing the planter to vibrate. This results in poor performances of the seed discharge by seed discharger and the seed guide by the seed guide tube. Based on the principle of spiral conveying, a soybean shaftless spiral seed discharge and seed delivery device was designed. The optimum seed filling size and speed range of the spiral blade were obtained by analyzing the size, force, and motion of soybean seeds of "ZhongHuang 37". The quadratic regression orthogonal rotation test and response surface method were used to analyze the operating parameters of the shaftless spiral seed discharge and seed delivery device by joint EDEM (Discrete Element Method)-RecurDyn simulation. The optimum parameters were obtained: the spacing of spiral was 11.4 mm, spiral outer radius was 5.5 mm, spiral inner radius was 2.9 mm and rotation speed was 10.4 r·s^−1^. Based on simulation and optimization results, the device was trialed and its field performance was tested. The results showed that at a surface slope of 16.1°, an average surface flatness of 8.9 cm, an average planter vibration frequency of 75.2 Hz, and an average amplitude of 7.2 mm, the average seeding qualification index, multiple index, missing seeding index, and damage index of the shaftless spiral seed discharge and seed delivery device were 92.6%, 5.03%, 2.4% and 0.92%, respectively, which were in line with the local agronomic requirements. The designed soybean shaftless spiral seed discharge and seed delivery device meets the requirements of the quality of no-till seeding and can provide a reference for the design and improvement of seed discharger and seed guide tube under poor ground leveling and long-distance seed delivery conditions.

## Introduction

The soybean production of China is ranked fourth in the world^1^, and the soybean planting area in Southwest China (Typical Karst Landform, hilly terrain) accounts for 10.7% of the country in 2022^2^. Most of the southwest regions are sloping land, and the amount of straw mulch is large^3^. When the driven stubble-breaking anti-blocking no-till planter^4^ works, the presence of straw and root stubble on the no-till surface leads to poor flatness. Additionally, significant vibrations occur when the stubble-breaking blade cuts through straw, soil, and root-soil complex. This significantly impacts the seed filling process, seed discharge, and the movement of seeds in the seed guide tube^5^. This can result in miss seeding as well as poor uniformity and consistency, leading to reduced crop yields^6^.Therefore, it is important to develop a seed discharge and a seed delivery device suitable for the driven stubble-breaking and anti-clogging no-till planter in Southwest China^6^.

At present, the seed metering device is generally divided into pneumatic and mechanical^7,8^. The pneumatic seed metering device is not suitable for no-till seeding in the Southwest China because of the complicated and high cost of the pneumatic mechanism^9,10^. Besides, the topography of the southwest is mostly hilly and mountainous, and it’s dusty when doing the no-till seeding operation, therefore, small machine is more suitable for the southwest region^11^. The mechanical soybean seed metering device is divided into nest-eye, groove, and finger -types, etc. Relevant researchers had continuously optimized and improved mechanical seed metering device^12^; Dun Guoqiang et al^13^ optimized the diameter, depth and chamfer length of the type hole of the seed discharge disc to derive the optimal seed filling parameters and improve the seed filling rate; Huang Yuxiang et al^14^ proposed a side-guided seed metering device, which improved the seed filling rate and the seeding qualification rate, and reduced the seed damage rate and the seed missing rate by guiding soybean seeds and shortening the seeding distance; Hou Shouyin et al^12^ proposed a flexible and mechanical soybean seed metering device with seed cleaning brushes as well as seed protection brushes to improve the seed cleaning rate and reduce the seed damage rate; Liu Hongxin et al^15^ designed an opposed swashplate soybean seed metering device for the problem of decreasing seed filling rate of seeding at high speed, which improved the seeding qualification rate by changing the seed filling angle and the seed filling force. All of the above studies solved the problems of seed filling, seed cleaning, andseed protection, and improved the qualified rate of seed discharge. However, the seed discharge condition of low constraint stability of transport caused by the seed discharge device and seed guide tube is not analyzed when the vibration is high. Previous studies have shown that vibration has a significant effect on seeding^16^. The vibration makes the trajectory of the seed in the seed guide tube produce irregular changes^17^, resulting in the irregular direction and size in the process of the seed casting, which in turn affects the quality of seeding. And with the increase in the speed of seeding, the amplitude will be increased^18^.

The vibration generated in this study is mainly due to the impact of the driven stubble breaking blades in contact with the ground while cutting the soil and straw stubble. To address the above problems, utilizing the constraints of the spiral conveyor and the seed delivery tube on the seed to solve the effects of vibration on seed discharge and throwing, a soybean shaftless spiral seed discharge and seed delivery device was designed based on the principle of spiral conveying. The spiral size of the shaftless spiral conveying device was designed by using the three-axis parameters of soybean seed; the mechanical analysis of seed filling, seed delivery and seed throwing of the device was conducted. The joint simulation of RecurDyn and EDEM flexible body was established, and the quadratic regression orthogonal rotation combination test was conducted to determine the optimal combination of parameters for the shaftless spiral seed discharging and conveying device. The field test was conducted with this trial device to verify the work reliability of the soybean shaftless spiral seed discharge and seed delivery device.

## Materials and methods

### Overall structure and working principle

#### Overall structure

**Figure 1:**
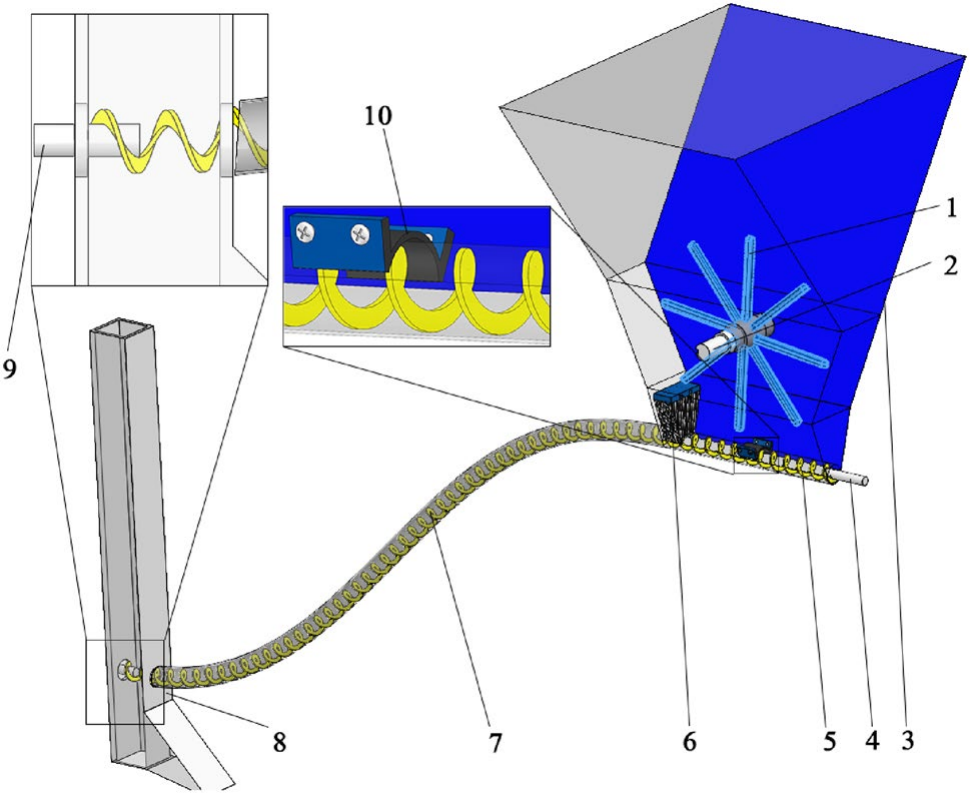
Schematic diagram of the structure of soya bean shaftless spiral seed discharge and seed delivery device. (1) Gearing, (2) gearing drive shaft, (3) seed box, (4) shaftless spiral blade rotation shaft, (5) shaftless spiral blade, (6) brush, (7) seed delivery tube, (8) trencher, (9) shaftless spiral blade fixed shaft, (10) elastic rubber belt.

Soybean shaftless spiral seed delivery device as shown in Fig. 1. The device parameters are shown in detail in Table 1.

**Table 1:** Device parameters.

Name	Material	Specification
Gearing	ASTM304	Diameter 6 mm
Gearing drive shaft	C45	Diameter 15 mm
Seed box	ASTM304	Thickness 0.5 mm
Shaftless spiral blade rotation shaft	C45	Diameter 6 mm
Shaftless spiral blade	65 mn	3350 mm × 1 mm × 2.6 mm
Brush	Nylon (loanword)	30 mm × 25 mm × 20 mm
Seed delivery tube	Polyurethane PU pipe	Length 960 mm and inner diameter 12.5 mm
Shaftless spiral blade fixed shaft	C45	Diameter 6 mm

The device mainly consists of gearing, gearing drive shaft, seed box, shaftless spiral blade rotation shaft, shaftless spiral blade, brush, seed tube, trencher, shaftless spiral blade fixed shaft, elastic rubber belt and so on. One end of the seed delivery tube is fixed to the seed box, and the other end is fixed to the furrow opener. The front part of the shaftless spiral blade is placed in the seed box, the middle part is placed in the seed delivery tube, and the end part is placed in the empty cavity of the furrow opener. And both sides of the shaftless spiral blade are connected to the shaftless spiral blade rotation axis and the shaftless spiral blade fixed axis. The brush is placed on the side of the seed box connected to the seed delivery tube. The elastic rubber band is fixed on both sides of the seed box, coaxial to the shaftless spiral blade, and it is placed in the middle of the plane between the brush and the seed box. The gearing is placed in the middle of the seed box.

#### Working principle

**Figure 2:**
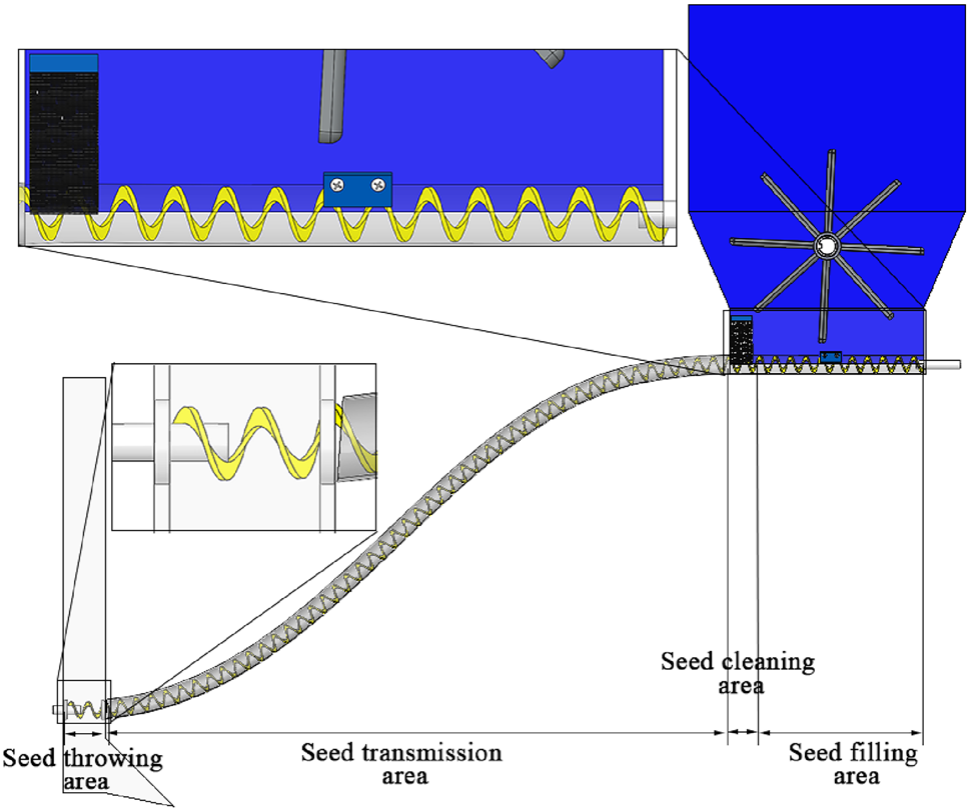
Diagram of the working of the shaftless spiral seed discharge and seed delivery device.

The working process of the soybean shaftless spiral seed discharge and seed delivery device is divided into four stages: seed filling, seed cleaning, seed transmission and seed throwing, as shown in Fig. 2. The power drives the shaftless spiral blade rotation shaft and the gear transmission shaft, and the two shafts drive the shaftless spiral blade and gear rotation respectively when working. The elastic rubber band restrains the shaftless spiral blade to ensure that the axis position does not deviate, and the shaftless spiral blade will continuously rotate the soybean seeds that enter the spiral space from the filling area to the clearing area. When seeds pass through the brush, the excess seeds in the spiral pitch will be combed off. At the same time, the paddle teeth rotate counterclockwise to paddle the seeds in the seed box to prevent the seeds from emptying due to the narrow space, which affects seed filling. After seeds passing through the brush and enter the seed transmission stage, the seeds are pushed by the shaftless spiral blades to move in the seed deliverytube. As the seeds are bound by the seed transmission tube in a small space, the seeds move according to the movement trajectory until they reach the empty cavity of the furrow opener. When the seeds enter the cavity of the seed opener, they are released from the shaftless spiral blades and move in a parabolic motion until they fall to the ground, as they lose the restraint of the seed delivery tube and are only subject to gravity. The seed delivery tube can be mounted on the furrow opener at the lowest position from the ground according to the requirements of different planting depths, minimizing the height of seed throwing.

### Design of key components

#### Structure and parameter design of shaftless spiral blade

When seeds are filled, their filling angle, attitude, and arrangement state will directly affect the filling performance^19^. The shape and size of the seed delivery tube will directly affect the movement trajectory of the seeds when seeds move in the seed delivery tube ^5^, so the design of the seed discharge device as well as the seed delivery device is of paramount importance.

**Figure 3:**
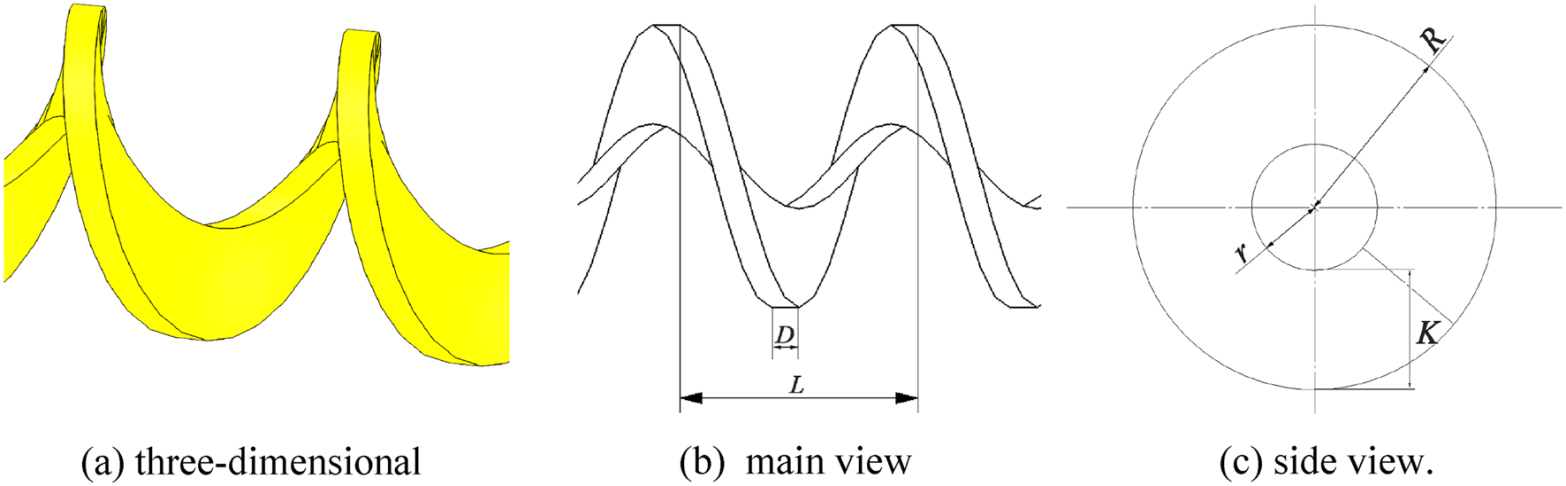
Schematic diagram of the structure of the shaftless spiral blade. L is the spiral pitch; D is the thickness of the spiral blade; r is the spiral inner radius; R is the spiral outer radius; K is the height of the spiral blade.

Due to the high sphericity of soybean seeds^20^ and their good rollability and mobility, a shaftless spiral seed discharging and delivery device was designed based on the principle of spiral conveying. Using its rotational pushing principle and the flexibility of the shaftless spiral, the seed discharging and the restrained seed delivery of soybean seeds were realized, and the structure schematic is shown in Fig. 3. In order to effectively enhance the capacity of seed filling and delivery, the spiral pitch, spiral inner radius, and spiral outer radius structural dimensions were designed based on the three axes of soybean seed dimensions.

##### Basic parameters of soybean seeds

The test was conducted using the soybean variety "Zhonghuang 37". 1000 soybean seeds were randomly selected and their length/*l*, width/*d* and thickness/*h* were measured with the parameters shown in Table 2.

**Table 2:** Three-dimensional parameters of soybean thousand seeds.

Seed parameters	Length (*l*)	Width (*d*)	Thickness (*h*)
Maximum value	10.25	8.1	7.18
Minimum value	7.12	6.06	4.91
Average	8.5507	7.3295	6.2605

The measured seed weight per thousand was 271.1 g, the average sphericity was 85.58%, and the seed size distribution followed the standard normal distribution^21,22^.

##### Design of shaftless spiral blade structure

When filling the seeds of soybeans, a filling quantity of 1 grain between spirals is appropriate in order to ensure that the subsequent seeding index is qualified. ^23^. It should be ensured that a single seed can be filled in every attitude, and the seeds are not affected by vibration and slope so that they are maximally constrained. According to Table 1, the average thickness of soybean seeds is ${\text{h}}_{\stackrel{\mathrm{-}}{\text{x}}}$= 6.26 mm, and twice the average thickness of the seeds is greater than the maximum length of the seeds ( ${\text{l}}_{\text{max}}$=10.25 mm). So the spiral pitch* L* size needs to be larger than the maximum length of the seed ${l}_{max}$ and less than twice the average thickness of the seed ${h}_{\overline{x} }$ in order to allow all sizes of seeds to be filled and only one seed to be filled between the spirals; When the shaftless spiral pushes the seeds, it must ensure that the seeds cannot pass through the shaftless gap to ensure the seeding quality, and its maximum diameter needs to be smaller than the minimum seed width ${\text{d}}_{\text{min}}$; The spiral outer diameter is analyzed in the same way as the spiral pitch L. Due to the transport way of the shaftless spiral is rotating transport, the radial force is larger, therefore, the minimum size of the outer diameter of the spiral is 5 mm (2 mm larger relative to the shaftless radius) to ensure the strength, and the expression equation is:

1 $$\begin{array}{c}\left\{\begin{array}{c}{\text{d}}_{\text{min}} \, \le  \, {\text{L}} \, \le {2}{\text{d}}_{\stackrel{{-}}{\text{x}}}\\ {2}{\text{r}} \, \le {\text{ d}}_{\text{min}}\\ {10} \, \le \, {2}{\text{R}} \, \le \, {2}{\text{h}}_{\stackrel{{-}}{\text{x}}}\end{array}\right.\end{array}$$

In the formula, r is the spiral inner radius, mm; *R* is the spiral outer radius, mm.

The data of shaftless spiral blades were calculated by substituting the soybean seed size parameters into the system of Eq. (1). The spiral pitch *L* is 6 to 12.5 mm. The range is 0.1 to 3 mm because the shaftless radius *r* cannot be 0. Considering that the spiral pushing seeds may produce radial circular motion when two smaller seeds are filled, the outer spiral radius *R* ranges from 5 to 6.5 mm in order to minimize damage to the seeds. During seed transmission, seeds will be transported by shaftless spiral to the furrow opener, which is not straight in space and needs to have a certain flexibility in the axial direction, so the thickness of spiral blade *D* is selected as 1 mm. To ensure that the spiral blades push the seeds forward as far as possible without jamming and damaging the seeds,the spiral inclination of the blade is 0, that is, the blade is perpendicular to the axial direction.

##### Speed analysis of shaftless spiral seed discharge

To determine its optimal discharge seed delivery speed, the shaftless spiral conveying volume per unit time is analyzed, and the conveying volume per unit time *Q* formula^24,25^ is:

2 $$\begin{array}{c}{\text{Q}}{=}\frac{\pi \text{L}{{(}{2}{\text{R}}{)}}^{2}-{4}{\text{D}}{(}\text{R-r}{)} \, {\text{L}}_{\text{P}}}{4}{\text{n}} \rho \varphi  \end{array}$$

In the formula, *L*_*P*_ is the length of a pitch of the shaftless spiral blade, mm; *n* is the rotational speed of the shaftless spiral blade, r/min; *ρ* is the seed density, g·cm^-3^; *φ* is the filling factor.

From the formula (2), it is known that the conveying volume of per unit time *Q* is related to *L*, *D*, *R*, *r*, *L*_*P*_, *n*, *ρ* and *φ*. Since the shaftless spiral blade is used for seed discharge and seed delivery. The shaftless conveying volume per unit of time is only related to its rotation speed n, and the higher its rotation speed, the higher the conveying volume, when the number of seeds between the spiral pitch *L* is certain.

**Figure 4:**
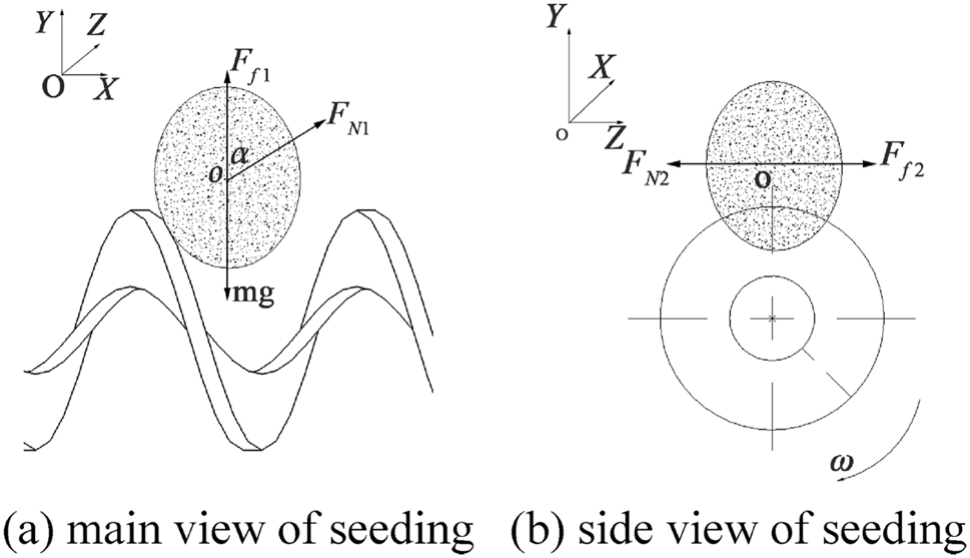
Schematic diagram of the vertical seed filling force.

When soybean seeds are filled between the spiral blades, the seeds are filled in various positions, such as vertically, diagonally and horizontally. While seeding, the spiral blades are also rotating, so the size of the spiral blade rotation speed affects the performance of soybean seeding. Since the vertical seeding and transverse seeding of soybean seeds are the longest and shortest seeding strokes in the horizontal and vertical direction, a mechanical analysis of soybean seeds is conducted for the vertical seeding case, and the force analysis is shown in Fig. 4.

According to Fig. 4, the mechanical equilibrium equation of the soybean seed with the spiral blade is as follows:

3 $$\begin{array}{c}\left\{\begin{array}{c}{\text{F}}_{{\text{f}} \, {1}}{+}{\text{F}}_{{\text{N}}{1}}{\text{cos}}\alpha {=}{\text{mg}}\\ {\text{F}}_{{\text{N}}{2}} {=}{\text{F}}_{{\text{f}} \, {2}}\end{array}\right.\end{array}$$

In the formula, *F*_*f* 1_ is the soybean seed and the vertical direction of the spiral blade friction, N; *F*_*N*1_ is the support force of the spiral blade on the soybean seed, N; ${\alpha }$ is the angle between *F*_*N*1_ and the vertical direction, °; *F*_*N*2_ is the support force of the soybean seed or the inner wall of the seed box on the seed, N; *F*_*f*2_ is the friction force of the soybean seed and the horizontal direction of the spiral blade, N.

As can be seen from the formula (3), when seeds in contact with the spiral blade below the center of mass, there is a balance between its gravity mg and friction force *F*_*f*1_ and support force *F*_*N*1_ in the vertical direction. The support force *F*_*N*2_ of soybean seeds or seed box inner walls on the seeds and the friction force *F*_*f*2_ of soybean seeds and the horizontal direction of the spiral blade balance. But the support force *F*_*N*1_ in the horizontal direction cannot be balanced, and the force point in the soybean seeds below the center of mass. Therefore, seeds will be separated from the spiral blade and upward movement. Through force analysis, seeds are supported by the spiral blade on its support force *F*_*N*1_ in the seed center of mass and above, and seeds are transported to the shaftless spiral blade under the force. Similarly, the horizontal seed filling also requires the spiral blade on its support force *F*_*N*1_ in the seed center of mass and above. Therefore, it needs to analyze the rotation speed of spiral blade to ensure that soybean seeds can enter the spiral blade smoothly.

**Figure 5:**
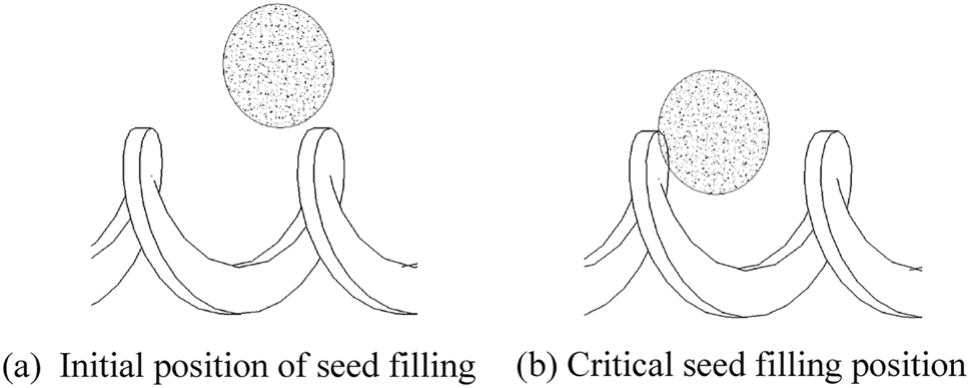
Optimal seed filling location for soybeans.

When the shaftless spiral blades are filled with seeds, they are constantly rotating, and the spiral pitch is constantly changing from side to side in relative position. The soybean seeds are arranged closely above the spiral, so there are always seeds in the optimal filling position (seeds are near the side that does not collide with the spiral blades), as shown in Fig. 5.

As can be seen from Fig. 5, the seeds do free fall motion and the shaftless spiral blades do rotational motion, whose equation of motion is:

4 $$\begin{array}{c}\left\{\begin{array}{c}\frac{\text{l}}{{2}}\text{=}\frac{1}{{2}}{\text{gt}}^{2}\\ {\text{L}} \, - \, {\text{L}}_{1}{=}{\text{d}}\\ {\text{L}} {\cdot }{\text{n}} {\cdot }{\text{t}} \, \le  \, {\text{L}}_{1}\end{array}\right.\end{array}$$

In the formula, L_1_ is the spiral pitch minus the remaining distance of the seed, mm; n is the speed of the shaftless spiral blade, r·min^−1^.

According to equation set 4, the maximum rotation speeds of the vertical and lateral seeding spiral blades of soybean seeds are 660 r·min^−1^ and 379.8 r·min^−1^, respectively. When the seeds are filled with the average size, the maximum rotation speeds of the vertical and lateral seeding are 860.4 r·min^−1^ and 711 r·min^−1^, respectively. According to the conventional speed of no-till seeding 0.56–1.39 m·s^−1^^26^, the seeding speed for soybean seeding at a seed spacing of 10 cm is:

5 $$\begin{array}{c}{\text{n}} {=}\frac{\text{vt}}{{\text{L}}_{\text{Z}}} \end{array}$$

In the formula, *L*_*Z*_ is the seeding spacing, cm; *v* is the planter forward speed, m·s^−1^; *t* is the time, s.

According to Eq. (5), the speed of the shaftless spiral blade is between 336 and 834 r·min^−1^. Since this is the most difficult seed filling condition, according to the average size of seed filling speed, the working speed of the shaftless spiral blade meets the conventional speed of no-till seeding, so the range of spiral blade seed filling speed is 336–834 r·min^−1^.

#### Design and mechanical analysis of the seed delivery tube

##### Design of seed delivery tube parameters

After filling the seed box, soybeans are driven by the shaftless spiral blades into the seed delivery tube, which makes sliding or rolling movements. The size and material of the seed delivery tube affect the trajectory and movement of soybeans. The seed tube and shaftless spiral blades need to fit closely due to the need to constrain the movement of soybean seeds. In order to facilitate installation, the inner diameter is 1 mm larger than the spiral blades, using a smooth and flexible inner wall and good wear resistance of polyurethane PU pipe.

##### Analysis of the coefficient of friction of the inner wall of the seed delivery tube

**Figure 6:**
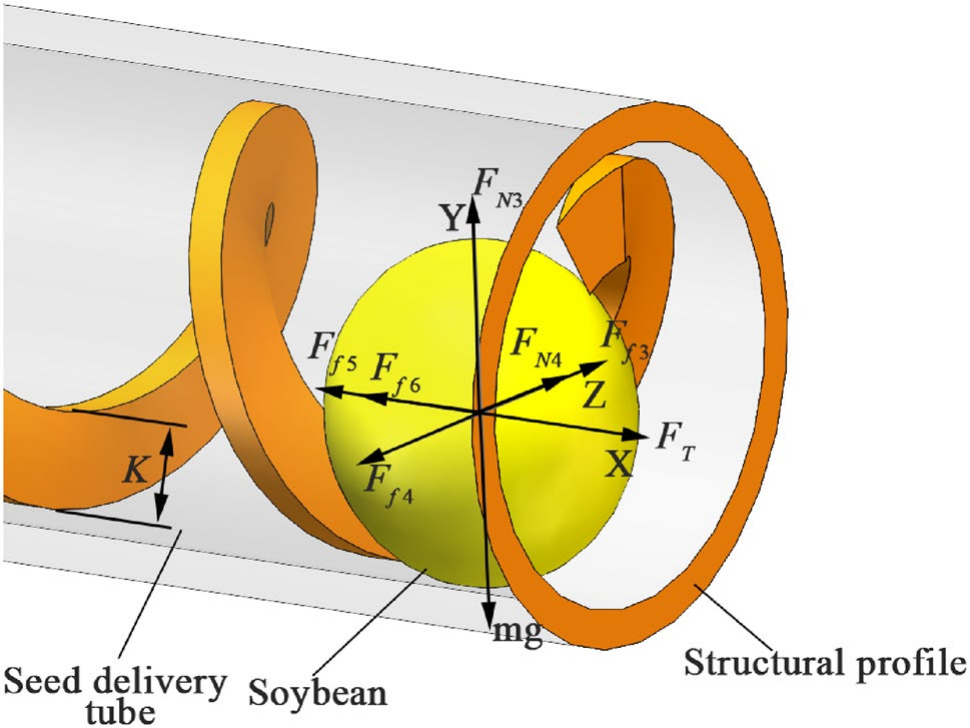
Schematic diagram of the forces on soybeans in the seed delivery tube. Spiral blade height *K* is 2–6.4 mm.

The movement trajectory of the soybeans is affected by the friction between the shaftless spiral blades, soybeans and the seed delivery tube. The seed mass center and the height K of the shaftless spiral blades affect whether the seeds will roll between the other spiral pitches, so a force analysis of the soybeans in the seed delivery tube is carried out, as shown in Fig. 6.

The friction coefficients of the seed delivery tube and the shaftless spiral blade against soybeans are divided into three cases. That is, the friction coefficient of the seed delivery tube on soybeans is less than, equal to or greater than the friction coefficient of the shaftless spiral blade on soybeans. When the coefficient of friction of the seed delivery tube to soybeans is less than the coefficient of friction of the shaftless spiral blade to soybeans, the set of equations is:

6 $$\begin{array}{c}\left\{\begin{array}{c}{\text{F}}_{{\text{N}}{3}} {=}{\text{mg}}\\ {\text{F}}_{{\text{f}}{3}} {+}{\text{F}}_{{\text{N}}{4}} {=}{\text{F}}_{{\text{f}}{4}}\\ {\text{F}}_{\text{T}} {=}{\text{F}}_{{\text{f}}{5}} {+}{\text{F}}_{{\text{f}}{6}}\end{array}\right.\end{array}$$

In the formula, *F*_*N*3_ is the support force of the seed delivery tube for the vertical direction of the soybean seed, N; *F*_*N*4_ is the support force for the seed delivery tube for the horizontal direction of the soybean seed, N; *F*_*f*3_ is the friction of the shaftless spiral blade on the soybean seed, N; *F*_*f*4_ is the radial horizontal friction of the bottom of the seed delivery tube on the soybean seed, N; *F*_*f*5_ is the axial horizontal friction of the bottom of the seed delivery tube on the soybean seed, N; *F*_*f*6_ is the axial horizontal friction of the side of the seed delivery tube on the soybean seed axial horizontal friction, N.

7 $$\begin{array}{c}{\text{K}} {=}{\text{R}}-{\text{r}} \end{array}$$

From the system of Eq. (6), it can be seen that the combined external force of soybean seeds is 0 N and soybean seeds move forward with uniform speed. Due to the presence of *F*_*f6*_, the soybean seed may make counterclockwise rotational motion in the Y-axis direction while advancing at a uniform speed, i.e., the soybean seed makes forward rolling motion against the side of the seed delivery tube. However, since the friction force of the shaftless spiral blade on the soybean seed may be greater than that of the seed delivery tube on the soybean seed, the seed may only advance at a uniform speed without rotational motion. In both cases, the soybean seed is conveyed smoothly. When the seed center of mass is higher than the height of the shaftless spiral blade, the friction coefficient of the seed delivery tube on soybean is smaller and it can still delivery seed normality.

When the coefficient of friction of the seed delivery tube on soybeans is equal to the coefficient of friction of the shaftless spiral blade on soybeans, the set of equations is:

8 $$\begin{array}{c}\left\{\begin{array}{c}{\text{F}}_{{\text{N}}{3}} \, {=} \, {\text{mg}}\\ {\text{F}}_{{\text{f}}{3}} \, {=}{\text{ F}}_{{\text{f}}{4}}\\ {\text{F}}_{\text{T}} \, {=} \, {\text{F}}_{{\text{f}}{5}}\end{array}\right.\end{array}$$

From the set of Eq. (8), the combined external force of soybean seeds is 0 N and soybean seeds move forward with uniform speed. However, when the seed’s center of mass higher than the height of the shaftless spiral blade, the seed may tilt and roll to the left spiral pitch, which causes miss of the spiral pitch, and multiple of the rest of the spiral pitch. Similarly, when the coefficient of friction of the seed delivery tube to soybeans is greater than the coefficient of friction of the shaftless spiral blade to soybeans, it may also cause seed leakage and multiple between spiral pitch.

When seeds are transported from the shaftless spiral blade to the seed delivery tube, the friction coefficient of the bottom of the seed box on the soybean seeds is different from that of the seed tube connection. When the friction coefficient of the seed delivery tube on soybean seeds is greater than that of the shaftless spiral blade on soybean seeds, it will cause seed bouncing and returning to the seed box situation. Comprehensive consideration, the friction coefficient of the seed tube to soybean seed needs to be less than the friction coefficient of the shaftless spiral blade on soybean seed.

#### Seed delivery tube curvature

**Figure 7:**
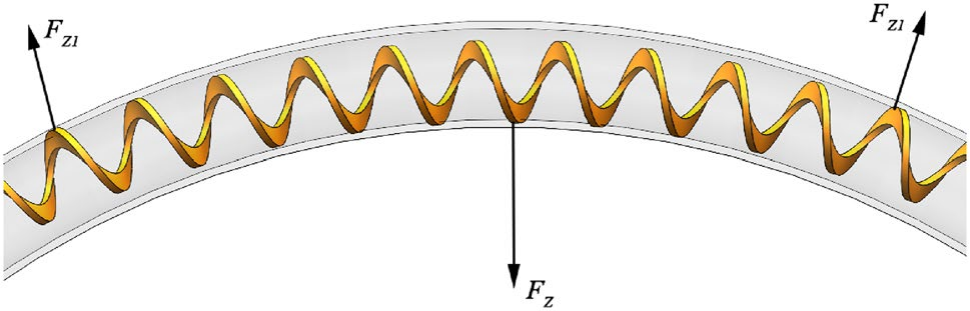
Schematic of contact forces. *F*_*Z*_ is the contact forces; *F*_*Z1*_ is half the contact forces.

The shape and curvature of the spatial arrangement of the seed delivery tube affect the friction between it and the shaftless spiral blade, which in turn affects the torque of the shaftless spiral blade during its operation. In order to ensure the normal operation of the shaftless spiral blade, the curvature of the seed tube is analyzed. When the seed tube is bent, the contact force between the shaftless spiral blade and the seed tube is generated by three contact points, i.e., the pivot points on both sides of the arc and the apex in the middle of the arc, as shown in Fig. 7.

As the spiral blade cross-section is rectangular, for the convenience of calculation, according to the cross-sectional area of the same rectangular body, the force exerted on it in bending is^27,28^ :

9 $$\begin{array}{c}{\text{F}}_{\text{Z}} {=}\frac{\text{48wEI}}{{\text{l}}_{\text{z}}^{3}}\end{array}$$

The expressions for the parameters *w* and *I* in the equation are:

10 $$\begin{array}{c}{\text{w}} {=}\frac{{{2}{\text{l}}}_{\text{z}}}{\pi}-\frac{{\sqrt{2}{\text{l}}}_{\text{z}}}{\pi} \end{array}$$

11 $$\begin{array}{c}{\text{I}}{=}\frac{{\text{D}}{({\text{R}}-{\text{r}})}^{3}}{12}\end{array}$$

In the formula, *F*_*Z*_ is the force to bend the spiral blade, N; *w* is the deflection, mm; E modulus of elasticity, Pa; *I* is the moment of inertia of the section, mm^4^; *l*_*Z*_ two pivot points spacing, mm.

Since the bending force of the shaftless spiral blade is generated by the seed delivery tube, the radial friction between the shaftless spiral blade and the seed delivery tube is:

12 $$\begin{array}{c}{\text{F}}_{\text{f }{7}} {=}{2}{\mu}{\text{F}}_{\text{Z}}\end{array}$$

In the formula, *F*_*f*7_ is the friction force between the shaftless spiral blade and the seed delivery tube, N.

Since the shaftless spiral blade is machined from 65Mn steel, its characteristics are similar to those of a spring of rectangular cross-section, which is subjected to a maximum torque of^29,30^:

13 $$\begin{array}{c}{\text{T}}_{\text{max}}{=}\frac{{\text{0.6}{\sigma}}_{\text{b}}{\text{D}}{\left({\text{R}}-{\text{r}}\right)}^{2}}{{6}{\text{K}^{\prime}}} \end{array}$$

In the formula, *T*_*max*_ is the maximum torque allowed without plastic deformation of the shaftless spiral blade, N m; *σ*_*b*_ is the tensile strength, Pa.

Where the parameter *K'* is expressed as:

14 $$\begin{array}{c}{\text{K}}{\prime} {=}\frac{{3}{\text{C}}-{1}}{{3}{\text{C}}-{3}}{=}\frac{{3}\frac{{\text{D}}_{1}}{\left({\text{R}}-{\text{r}}\right)}-{1}}{{3}\frac{{\text{D}}_{1}}{\left({\text{R}}-{\text{r}}\right)}-{3}} \end{array}$$

In the formula, *K′* is the curvature coefficient; *C* is the spin ratio; *D*_1_ is the spiral blade mid-diameter, mm.

To ensure that the shaftless spiral blades can work properly, the equation is:

15 $$\begin{array}{c}{\text{T}}_{\text{max}}{>}{2}{\mu}{\text{F}}_{\text{Z}}{\cdot }{\text{N}}_{1}\end{array}$$

In the formula, *N*_1_ is the total number of bending of the shaftless spiral blades.

In order to meet the requirement of the installation length of the seed delivery tube, the length of the seed delivery tube for small and medium-sized seeders is generally 1 m^31^, so the length of the seed delivery tube is adopted as 1 m. For the sake of calculating the number of bendable arcs and the radius size of the seed delivery tube, the length of the seed delivery tube is divided equally and the 90° arc bending is performed. The modulus of elasticity of 65Mn is 1.97 × 10^11^ Pa, and the friction coefficient of μ is 0.3. According to the derivation of formulas (9)–(15), the seed pipe can be bent at most three arcs of 90°, and the radius of the seed pipe is 43.4 mm.

#### Seed box design

**Figure 8:**
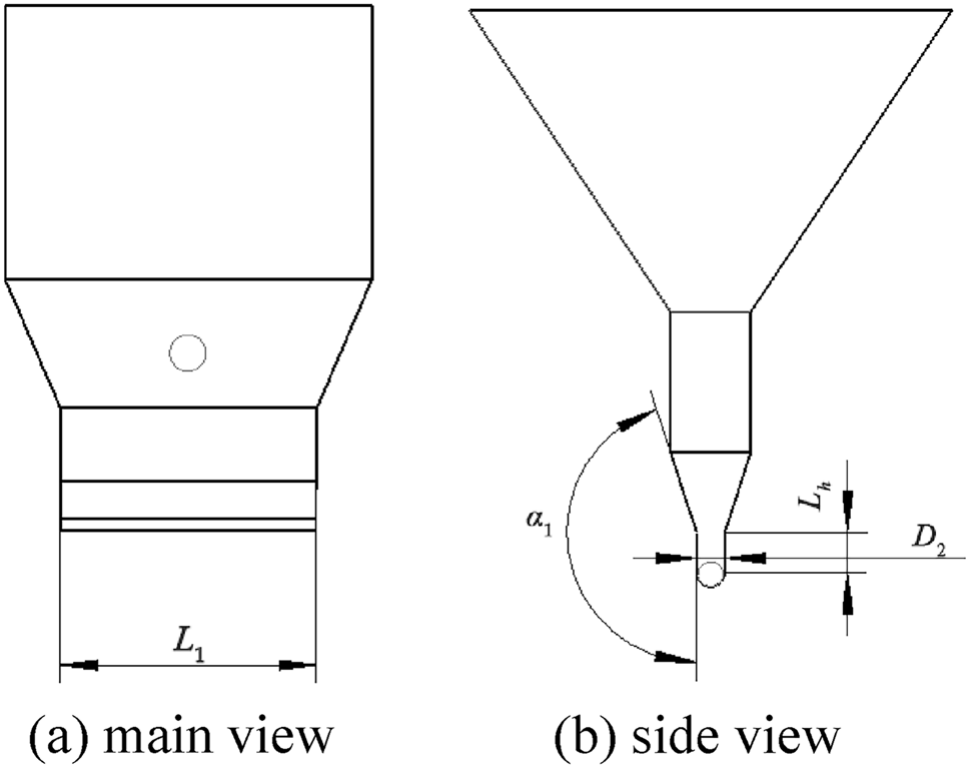
Schematic diagram of seed box structure.

To prevent the seeds filled into the spiral pitch from detaching due to vibration, it is necessary to limit the space for the seeds to move on both sides of the shaftless spiral blades. To ensure the contact between the shaftless spiral blade and the bottom of the seed box, the distance between the inner wall of the seed box and the spiral blade should be minimized, as shown in Fig. 8 (the figure shows the size of the inner wall of the seed box).

To prevent friction between the shaftless spiral blades and the seed box, the spacing is 0.5 mm, the formula is:

16 $$\begin{array}{c}{\text{D}}_{2}{=}{2}{\text{R}}{+}\text{0.5}\end{array}$$

In the formula, *D*_2_ is the width of the bottom seed box, mm.

In order to facilitate seed filling, a seed filling zone of *L*_*h*_ size is added in the vertical direction of the shaftless spiral blade. The height of the zone is the maximum length of two seeds, which is 20 mm. To ensure that the seed slides down to the seed filling zone on the inner wall of the seed box, it is necessary to make the tilt angle of the inner wall of the seed box greater than the friction angle. According to the static friction coefficient of the seed box to soybean seed is 0.4^32^ the formula obtained:

17 $$\begin{array}{c}{\alpha}_{\text{z}}{=}{\alpha}_{1}-{90}{\circ} \ge  {\text{arctan}}{0.4} \end{array}$$

In the formula, *α*_*z*_ is the angle at the inclination of the seed box °.

It is known from Eq. (17) that *α*_1_ should be greater than 112°. For good seed filling of soybean seeds in the seed box, the length of the spiral blade in the seed box should be as long as possible. However, it will lead to poor fit of the spiral blade and the inner wall of the seed box if the value is too large, so the length of *L*_1_ is:

18 $$\begin{array}{c}{\text{L}}_{1} {=}{10}\left(\text{L+D}\right){+}{\text{L}}_{\text{m}} \end{array}$$

In the formula, *L*_*m*_ is the brush width, mm.

### Simulation test

In order to investigate the optimal working conditions for seed discharge and delivery of the shaftless spiral blades, simulation tests were conducted to determine the optimal parameters for the spiral pitch, spiral outer radius, spiral inner radius and spiral blade rotation speed.

#### Simulation model building

Since the shaftless spiral blade is a flexible body, the EDEM-RecurDyn flexible body coupling simulation is used for a more accurate analysis of the working parameters. In order to investigate the best seed filling, seeding performance and reduce the computational effort of the simulation software, the simulation was carried out with the shaftless spiral blade and the seeding tube for horizontal seed discharge and delivery.

**Figure 9:**
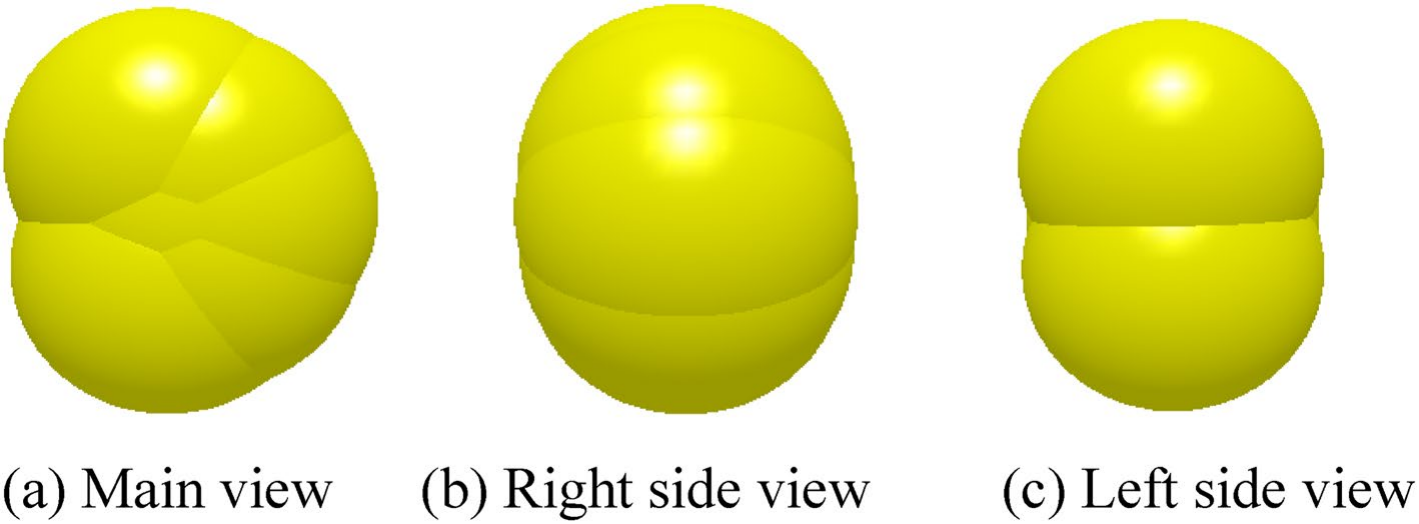
Soybean seed simulation model.

The EDEM software was used to model the soybean seeds with the average triaxial dimensions of the seeds, as shown in Fig. 9. The relevant literature^29^ was checked with the friction angle method^33^ to determine the intrinsic parameters of the soybean, 65Mn, and polyurethane PU tube and the contact parameters between phases, as shown in Table 3.

**Table 3:** Discrete element simulation parameter model.

Project	Parameter	Value
Soybean seeds	Poisson's ratio	0.25
	Shear modulus (Pa)	1.08 $\times$ 106
	Density (kg m^−3^)	1216
65Mn	Poisson's ratio	0.3
	Shear modulus (Pa)	8 $\times$ 1010
	Density (kg m^−3^)	7800
Polyurethane PU pipe	Poisson's ratio	0.38
	Shear modulus (Pa)	2.5 $\times$ 106
	Density (kg m^−3^)	1160
Soybeans–soybeans	Recovery coefficient	0.58
	Static friction factor	0.44
	Dynamic friction factor	0.05
Soybean–polyurethane PU pipe	Recovery coefficient	0.56
	Static friction factor	0.29
	Dynamic friction factor	0.01
65Mn–soybeans	Recovery coefficient	0.6
	Static friction factor	0.4
	Dynamic friction factor	0.02
65Mn-polyurethane PU pipe	Recovery coefficient	0.5
	Static friction factor	0.3
	Dynamic friction factor	0.2

**Figure 10:**
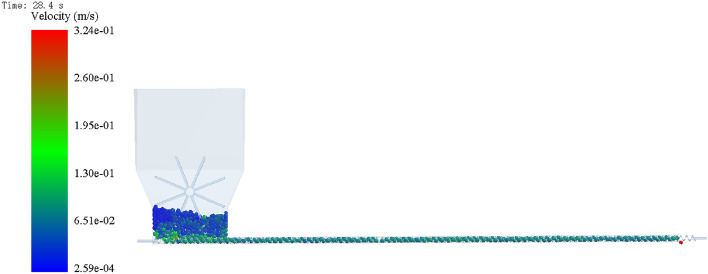
Simulation model of shaftless spiral seed discharge and seed delivery device.

To ensure the accuracy of the test, soybean seeds were generated with a normal distribution size^34^, and the particle factory was used to generate a mass of 0.5 kg of soybean seeds within 1 s. After 1 s, the shaftless spiral blades and the paddle teeth started to rotate, and the rotation sub was set by RecurDyn. Since the paddle teeth only disturb the seeds to prevent causing empty cavities, the rotation speed is 10 r·min^-1^, and the speed and parameters of the shaftless spiral blade are set according to the test program. The generated wall file was imported into EDEM, and the simulation model is shown in Fig. 10. When seeds are discharged, count the number of seeds discharged per revolution from the first discharged seed until that the shaftless spiral blade has made 300 revolutions, and repeat three times for each group.

#### Experimental factors and experimental indicators

From the previous theoretical analysis, it is known that the seed discharge quality of the shaftless spiral seed delivery device is related to the spiral pitch, spiral outer radius, spiral inner radius and rotation speed. To investigate the optimal operation parameters, a simulation test was carried out to analyze the four influencing factors. The spiral pitch, spiral outer radius, and spiral inner radius were a range of 6–12.5 mm, 5–6.5 mm, and 0.1–3 mm, respectively. The range of the spiral rotation speed was 0.56–1.39 m·s^−1^ according to the conventional speed of no-till seeding^24^, that is, 336–834 r·min^−1^. In order to ensure the accuracy of the rotation speed, the rotation speed range was expanded to 300–840 r·min^−1^ (5–14 r·s^−1^). The test indexes were used for seed filling rate (1–2 seeds/one turn of spiral blade rotation), multiple rate (> 2 seeds/one turn of spiral blade rotation), and missing rate (0 seeds/one turn of spiral blade rotation)^35^, that is, the number of soybean seeds when the seeds were discharged from the seed delivery tube.

A four-factor, five-level quadratic regression rotated orthogonal combination test (1/2 implementation) was conducted for the influencing factors, and the test factors and levels are shown in Table 4.

**Table 4:** Factors and levels of the experiment of shaftless spiral seed discharge and seed delivery device.

Level	Spiral pitch *X*_1_ (mm)	Spiral outer radius *X*_2_ (mm)	Spiral inner radius *X*_3_ (mm)	Rotation speed *X*_4_ (r·s^−1^)
1.682	12.5	6.5	3.00	14.0
1	11.2	6.2	2.41	12.2
0	9.25	5.75	1.55	9.5
−1	7.3	5.3	0.69	6.8
−1.682	6	5	0.10	5.0

### Field trials

#### Test conditions and equipment

The experiment was conducted at the conservation tillage experimental field of Kunming University of Science and Technology (N 24°50′56″, E 102°51′49″) on April 28, 2023. The test field was 50 m long and 20 m wide, with an average soil moisture content of 21.94% in the 0–15 cm soil layer, predominantly sandy clay loam and sandy clay soil texture, average soil bulk weight of 1.24 g·cm^−3^, average soil firmness of 1210 kPa, surface slope of 16.1°, and average surface unevenness of 8.9 cm. The average daily temperature of the test was 24 °C, with no rainfall, maximum wind speed of 2.0 m·s^−1^, and average wind speed of 1.43 m·s^−1^. The previous crop was corn, the straw moisture content was 9.3%, the mulching rate was 93%, and the mulching volume was 1.43 kg·m^−2^. The state of the corn straw was not crushed, and the whole straw was mulched.

The combination of a shaftless spiral seed discharge and delivery device was carried out according to the optimal combination of parameters. The test was conducted using "Zhong Huang 37" soybean seed as the test object. And the test supporting device was a active stubble-breaking and anti-blocking no-till planter with forward-reverse rotation powered by a Dong Fang Hong 500 tractor^3^. Three types of commercially produced seed dischargers are used: spoon-type seeder, finger-type seeder and external grooved wheel seeder. When working, the average frequency of vibration of the active stubble-breaking and anti-blocking no-till planter with forward-reverse rotation was measured to be 75.2 Hz, and the average amplitude of vibration was 7.2 mm.

#### Test methods and evaluation indexes

**Figure 11:**
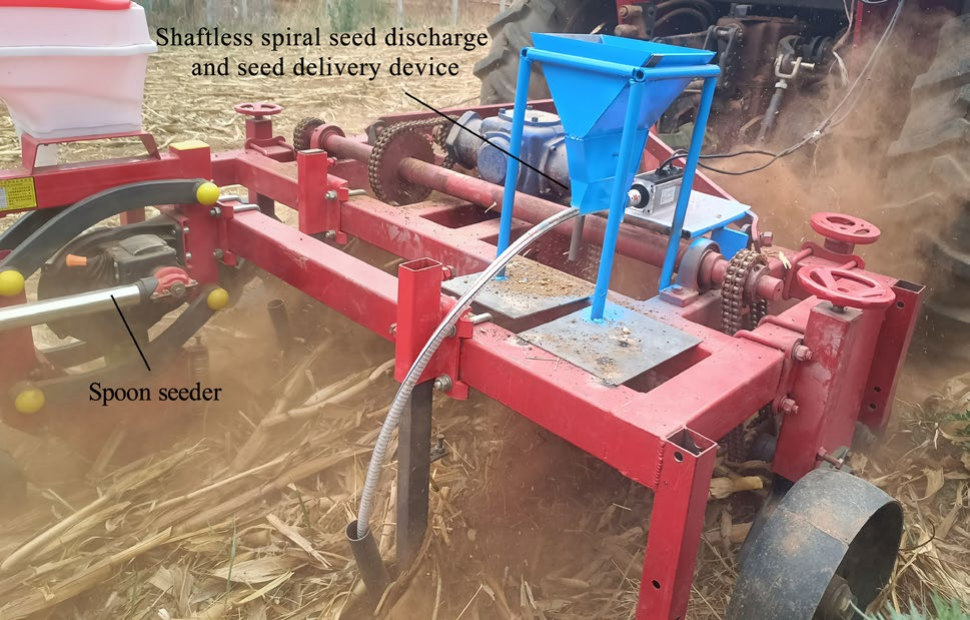
Field trial.

To test the seed discharge and delivery performance of the device, a field comparison test was conducted. The external grooved wheel seeder, finger-type seeder, spoon-type seeder and shaftless spiral seed discharge and delivery device are mounted on the frame of no-tillage planter in sequence, and seeding was performed at a forward speed of 1 m·s^−1^, as shown in Fig. 11. The test was conducted five times in the field, with each seeding length being 40 m.

After seeding, three sections randomly selected at 10 m intervals (except for a 10 m buffer zone before the start of seeding) were marked, and the topsoil was plucked away to expose the soybean seeds, the quality of seeding was measured. Each group of tests was repeated three times.

The evaluation indexes were selected according to the national standard "GB/T 20865-2017 No or little-tillage fertilizes-seeder"^36^ and "GB/T6973-2005 Testing methods of single seed drills(precision drills)"^37^.The qualification index y_1_, multiple index y_2_, missing index y_3_ and damage index P_1_ were selected as evaluation indexes, with the formula:

19 $$\begin{array}{c}\left\{\begin{array}{c}{\text{y}}_{1}{=}\frac{{\text{n}}_{1}}{{\text{N}}^{\prime}} \times {100}{\%}\\ {\text{y}}_{2} {=}\frac{{\text{n}}_{2}}{{\text{N}}{\prime}} \times {100}{\%}\\ {\text{y}}_{3} {=}\frac{{\text{n}}_{3}}{{\text{N}}{\prime}}  \times {100}{\%}\\ {\text{P}}_{1}{=}\frac{{\text{P}}_{\text{s}}}{{\text{P}}_{\text{w}}}\times {100}{\%}\end{array}\right. \end{array}$$

In the formula, *n*_1_ is the number of qualified seeds; *n*_2_ is the number of reseeded; *n*_3_ is the number of missed seeds; *N'* is the theoretical number of seed rows; *P*_1_ is the seed damage rate; *P*_*s*_ is the number of broken seeds; *P*_*w*_ is the total number of undamaged seeds.

Soybean seeds seeding, qualified seeding, and missed seeding standards are:

In the formula, *X*_*r*_ is the theoretical grain distance, 10 cm; *L*_*r*_ is the actual distance between adjacent seeds, mm.

20 $$\begin{array}{c}\left\{\begin{array}{c}{{\text{L}}_{\text{r}}{<}{0.5}{\text{X}}}_{\text{r}} \, \left({\text{y}}_{2}\right)\\ {\text{0.5}{\text{X}}}_{\text{r}}{<}{{\text{L}}_{\text{r}} {<}{1.5}{\text{X}}}_{\text{r}}\\ {{1.5}{\text{X}}}_{\text{r}}{<}{\text{L}}_{\text{r}} \left({\text{y}}_{3}\right)\end{array}\right.\left({\text{y}}_{1}\right)\end{array}$$

## Results and discussion

### Simulation test results

#### Analysis of experimental results and regression modeling

The simulation test protocol and results are shown in Table 5.

**Table 5:** Experimental scheme and results of shaftless spiral seed discharge and seed delivery device.

No	Spiral pitch (*X*_1_)	Spiral outer radius (*X*_2_)	Spiral inner radius (*X*_3_)	Rotation speed (*X*_4_)	Qualification rate (*Y*_1_)	Multiple rate (*Y*_2_)	Missing rate (*Y*_3_)
1	11.18	6.20	2.41	12.18	71.33	20.34	8.33
2	11.18	6.20	0.69	6.82	62.67	34.00	3.33
3	11.18	5.30	2.41	6.82	72.33	2.33	25.34
4	11.18	5.30	0.69	12.18	50.67	0.33	49.00
5	7.32	6.20	2.41	6.82	13.00	0.33	86.67
6	7.32	6.20	0.69	12.18	10.00	0.67	89.33
7	7.32	5.30	2.41	12.18	5.33	1.33	93.34
8	7.32	5.30	0.69	6.82	6.33	18.00	75.67
9	12.50	5.75	1.55	9.50	73.00	14.00	13.00
10	6.00	5.75	1.55	9.50	1.33	0.67	98.00
11	9.25	6.50	1.55	9.50	29.33	12.00	58.67
12	9.25	5.00	1.55	9.50	24.67	2.67	72.66
13	9.25	5.75	3.00	9.50	93.67	1.67	4.66
14	9.25	5.75	0.10	9.50	69.00	13.00	18.00
15	9.25	5.75	1.55	14.00	56.00	6.00	38.00
16	9.25	5.75	1.55	5.00	55.00	19.67	25.33
17	9.25	5.75	1.55	9.50	65.33	7.67	27.00
18	9.25	5.75	1.55	9.50	75.67	4.67	19.66
19	9.25	5.75	1.55	9.50	70.00	1.00	29.00
20	9.25	5.75	1.55	9.50	74.67	3.33	22.00
21	9.25	5.75	1.55	9.50	68.67	3.00	28.33
22	9.25	5.75	1.55	9.50	73.00	1.00	26.00
23	9.25	5.75	1.55	9.50	70.00	11.00	19.00

Design Expert software was used to analyze the test results, and the non-significant factors were excluded, and the ANOVA is shown in Table 5.

21 $$\begin{array}{c}\left\{\begin{array}{c}{\text{Y}}_{1}{=}\text{71.82}{+}\text{25.11}{\text{X}}_{1}{+}\text{5.4}{\text{X}}_{3}-\text{13.06}{\text{X}}_{1}^{2}-{16.65}{\text{X}}_{2}^{2}\\ {+}\text{2.56}{\text{X}}_{3}^{2}-{6.58}{\text{X}}_{4}^{2}\\ {\text{Y}}_{2}{=}{4.64}{+}{4.33}{\text{X}}_{1}{+}\text{3.59}{\text{X}}_{2}-{3.49}{\text{X}}_{3}-{4.03}{\text{X}}_{4}\\ {+}{8.75}{\text{X}}_{1}{{\text{X}}}_{2}{+}{2.78}{\text{X}}_{4}^{2}\\ {\text{Y}}_{3}{=}{23.55}-{29.43}{\text{X}}_{1}-{5.8}{\text{X}}_{2}{+}{5.15}{\text{X}}_{4}-{8.7}{\text{X}}_{1}{{\text{X}}}_{2}\\ -{4.21}{\text{X}}_{1}{{\text{X}}}_{3}{+}{12.23}{\text{X}}_{1}^{2}{+}\text{15.82}{\text{X}}_{2}^{2}-{3.39}{\text{X}}_{3}^{2}{+}{3.8}{\text{X}}_{4}^{2}\end{array}\right.\end{array}$$

where *X*_1_–*X*_4_ are the coded values.

It can be seen from the test results that the spiral pitch, outer shaft radius, shaftless radius and rotation speed have significant influence on the test results. Changes in the dimensions of spiral pitch, outer shaft radius and shaftless radius affect the changes in seed filling space, which in turn affects the seeding qualification rate, multiple rate and missing rate. Changes in rotation speed also led to changes in seed filling performance, which in turn led to changes in test results.

The variance results show that spiral pitch, outer shaft radius, shaftless radius and rotation speed have significant effect on the seeding qualification rate, multiple rate and missing rate. The interaction term between spiral pitch and outer shaft radius had a significant effect on multiple rate and leakage rate. The above regression model was tested for loss of proposed items. As shown in Table 6, the loss of proposed items were not significant (P > 0.05), indicating that the predicted values of the regression model fit the experimental values at a high level.

**Table 6:** Variance analysis.

Test index	Source	Sum of squares	Freedom	Mean square	*F* value	*P* value
Qualification rate (%)	Model	17,041.55	11	1549.23	49.08	< 0.0001
	*X*_1_	8608.35	1	8608.35	272.69	< 0.0001
	*X*_2_	66.68	1	66.68	2.11	0.174
	*X*_3_	398.91	1	398.91	12.64	0.0045
	*X*_4_	17.18	1	17.18	0.5443	0.4761
	*X*_1_* X*_2_	0.0144	1	0.0144	0.0005	0.9833
	*X*_1_* X*_3_	100.25	1	100.25	3.18	0.1023
	*X*_1_* X*_4_	10.13	1	10.13	0.3207	0.5825
	*X*_1_^2^	2710.34	1	2710.34	85.86	< 0.0001
	*X*_2_^2^	4407.11	1	4407.11	139.61	< 0.0001
	*X*_3_^2^	103.78	1	103.78	3.29	0.0972
	*X*_4_^2^	687.57	1	687.57	21.78	0.0007
	Residual	347.25	11	31.57		
	Loss of proposed items	268.41	5	53.68	4.09	0.0581
	Aberrant term	78.84	6	13.14		
	The sum	17,388.8	22			
Multiple rate (%)	Model	1589.62	11	144.51	17.69	< 0.0001
	*X*_1_	255.65	1	255.65	31.3	0.0002
	*X*_2_	176.1	1	176.1	21.56	0.0007
	*X*_3_	166.78	1	166.78	20.42	0.0009
	*X*_4_	221.34	1	221.34	27.1	0.0003
	*X*_1_* X*_2_	612.68	1	612.68	75.01	< 0.0001
	*X*_1_* X*_3_	3.58	1	3.58	0.438	0.5217
	*X*_1_* X*_4_	0.0561	1	0.0561	0.0069	0.9354
	*X*_1_^2^	10.98	1	10.98	1.34	0.2708
	*X*_2_^2^	10.98	1	10.98	1.34	0.2708
	*X*_3_^2^	10.98	1	10.98	1.34	0.2708
	*X*_4_^2^	122.43	1	122.43	14.99	0.0026
	Residual	89.85	11	8.17		
	Loss of proposed items	9.41	5	1.88	0.1403	0.9761
	Aberrant term	80.44	6	13.41		
	The sum	1679.47	22			
Missing rate (%)	Model	20,184.66	11	1834.97	45.4	< 0.0001
	*X*_1_	11,830.99	1	11,830.99	292.7	< 0.0001
	*X*_2_	459.52	1	459.52	11.37	0.0062
	*X*_3_	49.82	1	49.82	1.23	0.2906
	*X*_4_	361.86	1	361.86	8.95	0.0123
	*X*_1_* X*_2_	606.74	1	606.74	15.01	0.0026
	*X*_1_* X*_3_	141.71	1	141.71	3.51	0.088
	*X*_1_* X*_4_	8.67	1	8.67	0.2146	0.6522
	*X*_1_^2^	2376.27	1	2376.27	58.79	< 0.0001
	*X*_2_^2^	3978.1	1	3978.1	98.42	< 0.0001
	*X*_3_^2^	182.28	1	182.28	4.51	0.0572
	*X*_4_^2^	229.72	1	229.72	5.68	0.0362
	Residual	444.62	11	40.42		
	Loss of proposed items	341.31	5	68.26	3.96	0.0618
	Aberrant term	103.31	6	17.22		
	The sum	20,629.28	22			

Significant when 0.01 ≤ P < 0.05; extremely significant when P < 0.01.

#### Response surface analysis

**Figure 12:**
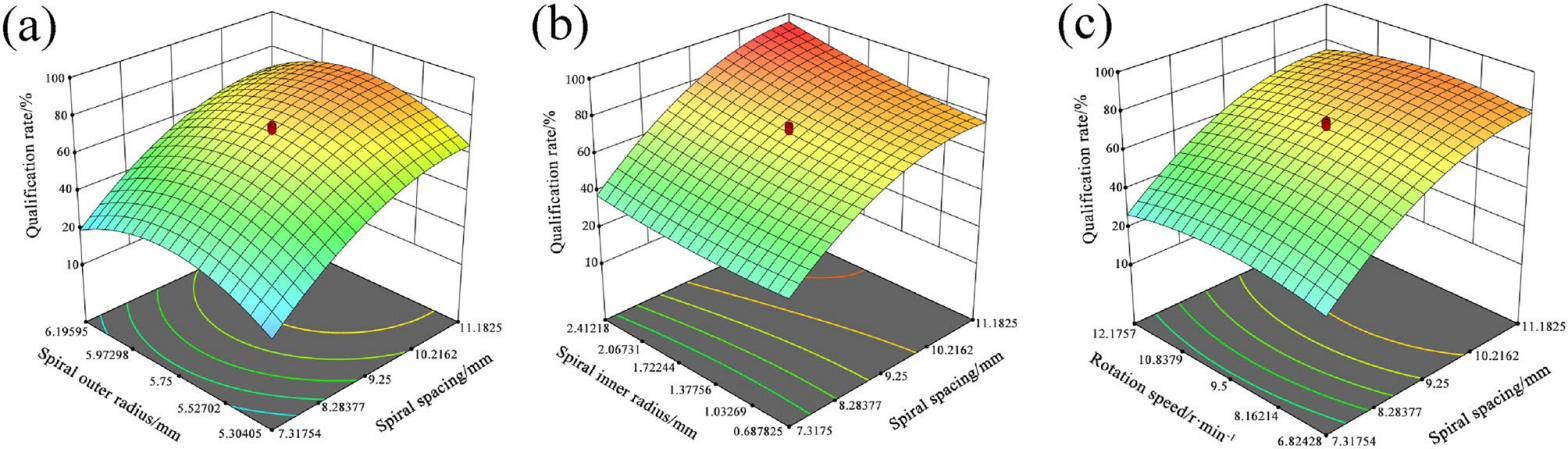
Effect of interactive factors on seeding pass rate. (**a**) Interaction between the spiral outer radius and the spiral pitch; (**b**) interaction between the inner of the spiral inner radius and the spiral pitch; (**c**) interaction between rotation speed and spiral pitch.

The surface response of the effect of interaction factors on the seeding pass rate was obtained using Design Expert software, as shown in Fig. 12.

The effect of the interaction term of the spiral outer radius and the spiral pitch on qualification rate is shown in Fig. 12a When the radius of the spiral outer radius in the initial value, the seeding qualification rate gradually increased with the increase in the spiral pitch, and the qualification rate was first increased and then decreased with the increase in the spiral outer radius. The reason for this phenomenon is that the spiral outer radius in the initial value and soybean seeds are easier filled between the spiral pitch due to the increase in the spiral pitch. When the spiral outer radius increases at the same time, the filling gap continues to expand, making it easier to fill the seeds. However, with both increasing too much, it leads to too much filling gap, making the multiple rate increase, which in turn leads to a reduction in the seeding qualification rate.

The effect of the interaction term of the shaftless radius and the spiral pitch on qualification rate is shown in Fig. 12b. The spiral spacing was positively correlated with the seed discharge qualification rate at the initial value of the shaftless radius, and the qualification rate further increased with the increase of the shaftless radius of the spiral. This indicates that when the shaftless radius is small, the seed filling gap is small and it is difficult for a small portion of seeds to enter the area between the seed filling gaps. As the shaftless radius increasse, the gap gradually increases and the qualifying rate of the seeding row also gradually increases.

The effect of the interaction term of the rotation speed and the spiral pitch on qualification rate is shown in Fig. 12c. The seed passing rate tended to increase with increasing spiral pitch at lower rotation speeds; however, the pass rate first increased slowly and then decreased sharply when the rotation speed increased. The reason for this phenomenon may be that when the rotation speed increases, more seeds have the opportunity to be filled, which results in a higher seeding rate. However, as the rotation speed increasing, the optimal rate of filling for most seeds is exceeded, resulting in a higher missing rate between filling gaps, which in turn decreases the qualification rate.

By analyzing the rotation speed, it can be seen that the influence of the changes of rotation speed of shaftless spiral blade on seed filling and seed discharge is slightly different from that of conventional seed discharger on seed filling and seed discharge. When filling and discharging seeds, with the increase of rotation speed, the effect of filling and discharging seeds of conventional seed discharger is getting worse and worse. But for the shaftless spiral blade, when working, with the increase of rotation speed, its seed qualification rate first increases and then decreases. The maximum rotation speed of the shaftless spiral blades during seed discharging is much higher than that of a conventional seed discharging disk (20–70 r/min).

The objective function is solved optimally with the constraints shown in Eq. (22).

22 $$\begin{array}{c}\left\{\begin{array}{c}{\text{max}}{\text{Y}}_{1}\left({\text{X}}_{1}{,}{\text{X}}_{2}{,}{\text{X}}_{3}{,}{\text{X}}_{4}\right)\\ {\text{Y}}_{2}\left({\text{X}}_{1}{,}{\text{X}}_{2}{,}{\text{X}}_{3} {,}{\text{X}}_{4}\right) \le {\text{minimum}}\\ {\text{Y}}_{3}\left({\text{X}}_{1}{,}{\text{X}}_{2}{,}{\text{X}}_{3}{,}{\text{X}}_{4}\right) \le {\text{minimum}}\\ {\text{s.t}}\left\{\begin{array}{c}{6} \, \le { \, {\text{X}}}_{1} \, \le  \, \text{12.5}\\ {5} \, \le \, {\text{X}}_{2} \, \le  \, \text{6.5}\\ \text{0.1} \, \le  \, {\text{X}}_{ 3} \le  \, {3}\\ {5} \, \le  \, {\text{X}}_{4 } \le  \, {14}\end{array}\right.\\ \end{array}\right.\end{array}$$

The optimal combination of the following parameters was obtained: the spiral pitch was 11.4 mm, the radius of the spiral outer was 5.5 mm, the radius of the spiral shaftless was 2.9 mm and rotation speed was 10.4 r·s^-1^. Under these conditions, the seeding rate was 96.54%, the multiple rate was 0.24% and the missing rate was 3.22%. According to the optimal parameters for the verification test, the seeding rate was 96%, and the relative error of the predicted value was 0.56%, which was consistent with the test value. The results can be used as the parameter of prototype production.

### Field trial results and analysis

**Figure 13:**
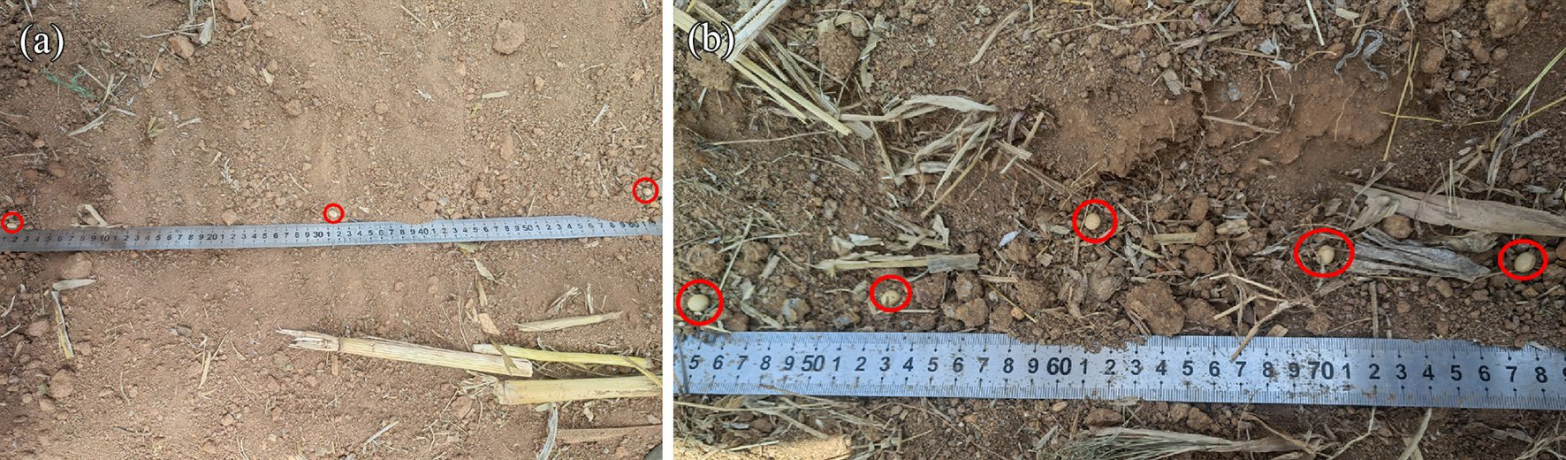
Comparison of seeding quality. (**a**) Seeding by spoon-type seeder; (**b**) seeding by shaftless spiral seed discharge and seed delivery device.

Since spoon-type seeder, finger-type seeder and external grooved wheel seeder are all commercially mass-produced seeders, seeding pass rates of about 90% can be achieved under normal seeding conditions. The performance of the different seeders can be determined by operating them at an average vibration frequency of 75.2 Hz and an average amplitude of 7.2 mm. The seeding effect of the field seeding test is shown in Fig. 13, and the results are shown in Table 6. As can be seen from Table 7, the average qualification index of spoon-type seeder, finger-type seeder and external grooved wheel seeder were lower than that of shaftless spiral seeder; while the multiple index and missing index were higher than that of shaftless spiral seeder. Among them, the spoon-type seeder had the lowest qualification index. During operation, due to that the spoon-type seeder has the least constraint on seeds, the seeds will be detached from the seed spoon causing missing when the machine vibrates and the slope is large. While the external grooved wheel seeder and finger-type seeder had higher constraints than the spoon-type seeder on seeds, so the qualification index was higher than that of the spoon-type seeder. As the shaftless spiral seed discharge and delivery device has the strongest constraint on seeds, its average qualification index, multipl index and misssing index were 92.6%, 5.03% and 2.4% respectively. And its seed damage index of 0.93% is lower than the metal material seed discharger seed injury standard (1.5%), in line with the seeding requirements. Under normal seeding conditions, the average vibration frequency is 20–50 Hz, and the average amplitude is 2–7 mm. When the vibration is stronger, the shaftless spiral seed discharge and delivery device is much higher than the other three seeders. And it meets the requirements of no-tillage seeding pass rate greater than 60% ^33^.

**Table 7:** Comparison test results between spoon-type seeder and shaftless spiral seed discharge and seed delivery device.

Seeding apparatus	Qualification index *y*_1_ (%)	Multipl index *y*_2_ (%)	Missing index *y*_3_ (%)	Damage index *P*_1_ (%)
Spoon-type seeder	38.5	1.6	59.9	0.62
	40.2	0.8	59	0.53
	37.7	2.38	59.92	0.75
Finger-type seeder	78.4	9.7	11.9	0.76
	78.6	9.1	12.3	0.84
	79.4	9.5	11.1	0.79
External grooved wheel seeder	72.4	13.6	14	0.56
	73.1	13.5	13.4	0.44
	72.5	14.1	13.4	0.48
Shaftless spiral seed discharge and seed delivery device	92.8	4.8	2.4	0.91
	93.7	4.4	1.9	1.01
	91.2	5.9	2.9	0.86

Note: results in three repetitions.

## Conclusions

The fields in Southwest China have a large amount of straw cover. When the the driven stubble-breaking and anti-blocking no-till planter is cutting straw, due to the poor flatness caused by straw and stubble on the surface of the no-tillage land, the machine tool is impacted to produce large vibrations. This will affect the working stability of the seed discharger to fill and discharge the seed, and the seed guide tube to guide the seed, resulting in the problem of poor seeding quality, leading to lower crop yields and reducing the economic benefits of crops. It is significant to solve problems of the stability of seed rowing, seed guide and improve the economic benefits of crops. Based on the principle of spiral conveying, the shaftless spiral blade was designed and the optimum design size of the shaftless spiral blade was analyzed by measuring the three shaft dimensions of soybean seeds. The force analysis of soybean seed filling was performed to derive the filling conditions to meet the soybean seed, and the speed range of the spiral blade filling was obtained by analyzing the optimal filling position. The relationship between the coefficient of friction of each contact part is obtained from the analysis of the friction between the shaftless spiral blade and the seed delivery tube on soybean seed. The maximum torque of the shaftless spiral blades and the friction between the blades and the seed delivery tube were analyzed to obtain the bending dimensions of the seed delivery tube installation.The EDEM-RecurDyn simulation model was established and a four-factor, five-level quadratic regression rotating orthogonal combination test was conducted to obtain the optimal parameters for the shaftless spiral seed discharge and seed delivery device. The device was prototyped with the optimal parameters, and field trials were conducted.

1. A shaftless spiral blade was designed by the principle of spiral conveying, and the design of dimensional parameters was completed. The mechanical and kinematic analyses were performed to reveal the seed-filling principle. The mechanical analysis of the shaftless spiral blade and the seed delivery tube was carried out to obtain the optimal installation dimensions.
2. Using EDEM and RecurDyn joint simulation test and according to the quadratic regression orthogonal rotation combination test and surface response analysis method, the spiral pitch of the shaftless spiral blade is 11.4 mm, the spiral outer radius is 5.5 mm, the spiral inner radius is 2.9 mm and the speed is 10 r min^-1^.
3. The field test results showed that when the vibration is stronger, the average quelification index, multiple index and missing index of the shaftless spiral seed discharge and delivery device are better than spoon-type seeder, finger-type seeder and external grooved wheel seeder. And its damage rate meets the national no-till seeding standard. It can solve the problems of high vibration and poor seeding quality when working with driven no-tillage seeder in Southwest China, and improve the efficiency of agricultural production.

The biggest difference between the shaftless spiral seed discharge and delivery device designed in this study and the traditional seed discharge and seeding devices is that it integrates seed discharge, delivery and casting. It has extremely higher seed constraints and more precise seeding accuracy, and it can adapt to the situation of high vibration and slope of seeding. The research can provide a reference for the design and improvement of seed discharger and seed guide tube under the conditions of high vibration of machine and long-distance seed delivery. Since there are many kinds of cash crops in the Southwest China, further research on the diversity of seed discharge of the shaftless spiral seed discharge device is needed to meet the seed discharge operation of many kinds of crops.

## Data Availability

The data used to support the findings of this study are included within the article.
